# Comparison of gene disruption induced by cytosine base editing‐mediated iSTOP with CRISPR/Cas9‐mediated frameshift

**DOI:** 10.1111/cpr.12820

**Published:** 2020-04-29

**Authors:** Lu Dang, Guanglei Li, Xinjie Wang, Shisheng Huang, Yu Zhang, Yuanxin Miao, Lisi Zeng, Shuzhong Cui, Xingxu Huang

**Affiliations:** ^1^ Affiliated Cancer Hospital & Institute of Guangzhou Medical University Guangzhou China; ^2^ School of Life Science and Technology ShanghaiTech University Shanghai China; ^3^ Shanghai Institute for Advanced Immunochemical Studies ShanghaiTech University Shanghai China; ^4^ Jingchu University of Technology Jingmen China; ^5^ CAS Center for Excellence in Molecular Cell Science Shanghai Institute of Biochemistry and Cell Biology Chinese Academy of Sciences University of Chinese Academy of Sciences Shanghai China

## Abstract

**Objectives:**

Recently developed CRISPR‐dependent cytosine base editor (CBE), converting four codons (CAA, CAG, CGA and TGG) into stop codons without DNA double‐strand breaks (DSB), serves as an efficient gene disruption strategy besides uncontrollable CRISPR‐mediated frameshift. However, the detailed difference of gene knockout between the two systems has not been clarified.

**Materials and methods:**

Here, we selected some sgRNAs with different position background, then HEK293T cells were transfected with CBE/Cas9 plasmids together with sgRNAs. GFP‐positive cells were harvested by fluorescence‐activated cell sorting (FACS) 48 hours after transfection. Genomic DNA was collected for deep sequencing to analyse editing efficiency and genotype. RNA and protein were extracted to analyse gene mRNA level using qPCR analysis and Western blot.

**Results:**

Here, we compared the gene disruption by CBE‐mediated iSTOP with CRISPR/Cas9‐mediated frameshift. We found BE‐mediated gene knockout yielded fewer genotypes. BE‐mediated gene editing precisely achieved silencing of two neighbouring genes, while CRISPR/Cas9 may delete the large fragment between two target sites. All of three stop codons could efficiently disrupt the target genes. It is worth notifying, Cas9‐mediated gene knockout showed a more impact on neighbouring genes mRNA level than the BE editor.

**Conclusions:**

Our results reveal the differences between the two gene knockout strategies and provide useful information for choosing the appropriate gene disruption strategy.

## 1 INTRODUCTION

CRISPR/Cas9 is now a powerful genome editing toolkit in gene modification for cells,^1^, ^2^, ^3^, ^4^ animals^1^, ^2^, ^3^, ^4^ and plants.^1^, ^2^, ^3^, ^4^ Non‐homologous end joining (NHEJ) following with CRISPR/Cas9‐mediated double‐strand break (DSB) can lead to the frameshifts, including introduction of insertions, deletions, translocations or other DNA rearrangements at the site of a DSB, and then result in gene knockout.^5^, ^6^, ^7^ But CRISPR/Cas9 gave rise to a significant increase in apoptosis,^8^ possibly owing to DSB‐induced toxicity.^9^, ^10^, ^11^ Furthermore, Grégoire Cullot and co‐workers found unexpected chromosomal truncations resulting from only one Cas9 nuclease‐induced DSB in cell lines and primary cells by a p53‐dependent mechanism.^12^ By analysing post‐transcriptional and post‐translational effects of frameshift‐inducing insertions or deletions (indels) in a panel of CRISPR‐edited cells lines, Lum, L. and his colleague observed changes in the array of transcripts or proteins expressed from CRISPR‐targeted genes in ~50% of the cell lines studied.^13^ Recently, cytosine base editor (CBE) mediates the direct conversion of a C•G base pair to T•A base pair, providing a new technique to precisely edit target genes.^14^ Moreover, CBE can install premature stop codons to disrupt genes by precisely converting four codons (CAA, CAG, CGA or TGG) into stop codons, which provides a new method for gene knockout without the potential side effects resulting from double‐strand DNA cleavage.^8^, ^15^

Although gene disruption has been achieved by CBE‐mediated iSTOP and CRISPR/Cas9‐mediated frameshift, the detailed difference of gene knockout between the two systems has not been clarified. Here, we selected 13 sgRNAs with different position background to thoroughly compare the two systems by analysing the genotype, gene expression using deep sequencing, qPCR and Western blot. We also detected the editing results using two adjacent sgRNAs.

## 2 MATERIALS AND METHODS

### 2.1 Cell culture and transfection

HEK293T cells were cultured in Dulbecco's Modified Eagle Medium (DMEM) (Hyclone, SH30243.01) supplemented with 10% foetal bovine serum (FBS) (v/v) (Gemini, 900‐108) and 1% penicillin streptomycin (v/v) (Gibco, 15140122). BE3 plasmid was obtained from Addgene (Addgene, 73021). sgRNA oligos were annealed into pGL3‐sgRNA‐EGFP expression vector with U6 promoter (Addgene, 107721). Transfection was performed according to the manufacturer's protocols (Thermo Fisher Scientific, 11668019). In brief, HEK293T cells were seeded on Poly‐L‐lysine solution (Sigma, P4707) coated 24‐well plates (JETBIOFIL, TCP010012), and transfection was performed at approximately 70% density about 14 hours after seeding, 333 ng sgRNA plasmids and 666 ng BE3/Cas9 plasmids were transfected with 2 μL Lipofectamine 2000 (Thermo Fisher Scientific, 11668019). The medium was replaced with fresh medium 6 hours after transfection, and GFP‐positive cells were harvested by fluorescence‐activated cell sorting (FACS) 48 hours after transfection. Cells were incubated at 37°C with 5% CO_2_. sgRNAs used are listed in Table S1.

### 2.2 Genomic DNA extraction and PCR amplification

Genomic DNA of cells collected by FACS was extracted using QuickExtract™ DNA Extraction Solution (Lucigen, QE09050) according to the manufacturer's protocols. All the primers used for PCR amplification can be found in Table S2.

### 2.3 Real‐time quantitative PCR (qPCR) analysis

Total RNA from knockout cell lines was isolated using the TRIzol reagent (Invitrogen, 15596018) and 1 μg of total RNA, in 20 μL mixed reverse‐transcription reagent, was reverse transcribed using HiScript II Q RT SuperMix with gDNA wiper (Vazyme, R223‐01). The specific primer pairs (Table S3) were used for qPCR amplification utilizing the ViiA™ 7 Real‐Time PCR System (Applied Biosystems). The qPCR reaction includes 1 μL of cDNA templates, 10 μmol/L of each specific primer and 10 μL 2 × ChamQ SYBR qPCR Master Mix (Vazyme, Q331‐02). The reaction procedure was set as follows: 95°C for 3 minutes and 40 cycles of 95°C for 15 seconds followed by 60°C for 1 minute. Relative quantification of the target gene expression was calculated using 2^−ΔΔct^ method. Each reaction was performed in triplicate, and the data were calculated as M ± SD.

### 2.4 Deep sequencing analysis

The primers for deep sequencing were listed in Table S4. The touchdown PCR reaction procedure was set as follows: 95°C for 3 minutes and 10 cycles of 95°C for 15 seconds, 68°C for 30 seconds followed by 72°C for 30 seconds; then 25 cycles of 95°C for 15 seconds, 58°C for 30 seconds followed by 72°C for 30 seconds.^16^ The purified PCR products were sequenced using Hiseq X‐10 (2 × 150) platform. The deep sequencing data were processed using BWA‐MEM algorithm. All the sequencing data were deposited in the National Omics Data Encyclopedia (NODE) under the project accession OEP000254.

### 2.5 Western blot analysis

Western blot analysis was performed according to the manufacturer's protocols. The used antibodies include anti‐p53 (Santa Cruz, sc‐126), anti‐APEX1 (Abcam, ab92744), anti‐APEX1 (CST, #4128), anti‐GAPDH (Abcam, Ab181602) and anti‐α‐tubulin (Abcam, Ab1825). Images were captured with Amersham Imager 600.

## 3 RESULTS

### 3.1 Both BE3 and CRISPR/Cas9 can effectively knock out the targeting genes

**FIGURE 1:**
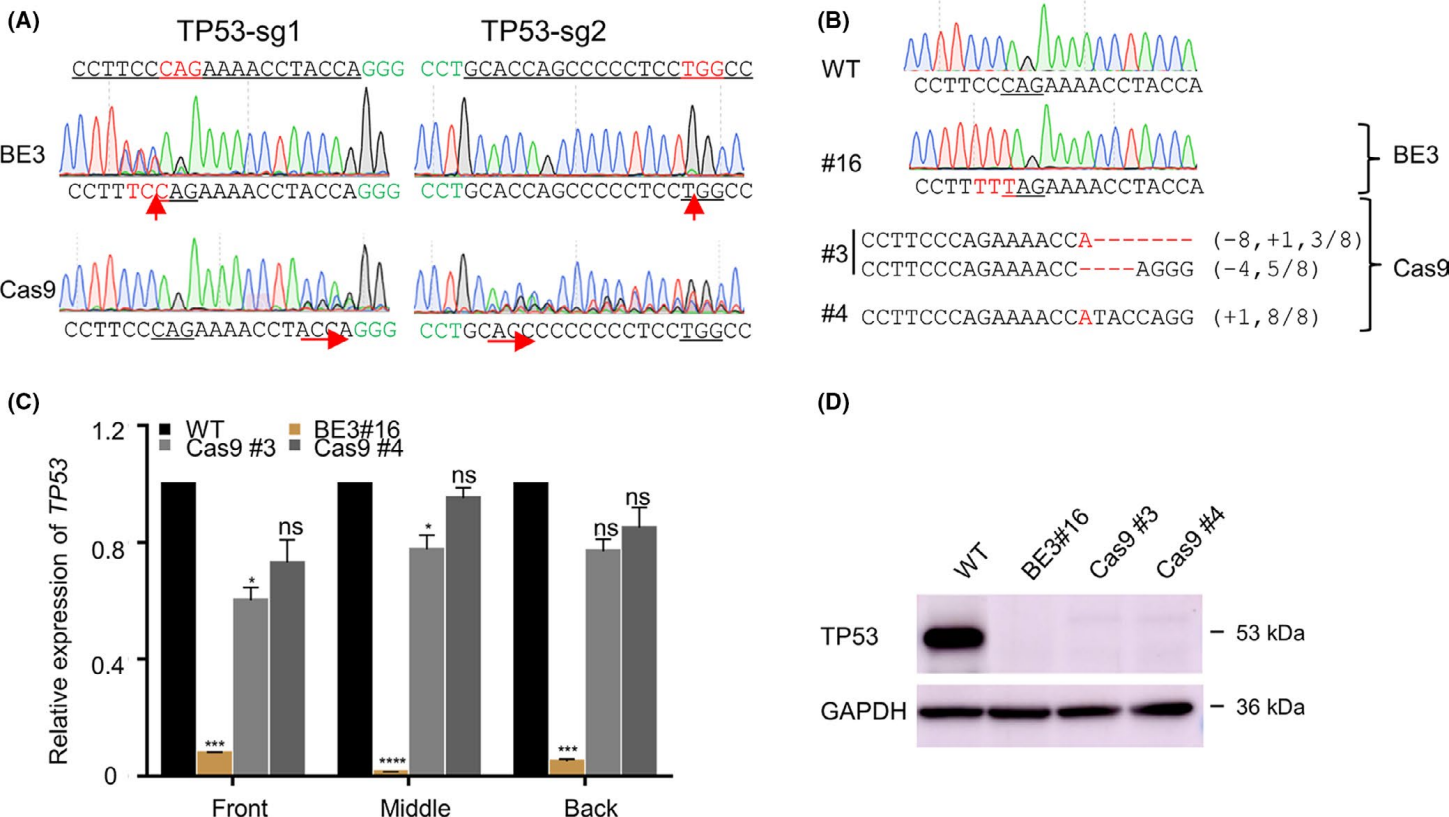
Both BE3 and CRISPR/Cas9 can effectively knock out the targeting genes. (A) The representative chromatograms of the Sanger sequencing for the target sites edited by BE3 or CRISPR/Cas9. PAM sequences are highlighted in green and sgRNA targeting sites are underlined. The red arrows show the modified sites. (B) The genotype of single‐clone cells for TP53‐sg1 edited by BE3 or CRISPR/Cas9. Stop codon sequences are underlined and the mutation in red. deletions (−), and insertions (+). (C) The analysis of the relative expression of TP53 detected with the qPCR assay. Front, middle or back mean the amplification region of qPCR relative to the sgRNA position. Statistical analyses highlighted the significant difference between WT clones (black) within each group. Data were analysed by Student's *t* test (**P < .01, ***P < .001, ****P < .0001, ns, not significant) and shown as means ± SEM (n = 3 from three independent experiments). (D) Immunoblotting of protein lysates from the knockout cell lines induced by BE3 and CRISPR/Cas9

Firstly, two sgRNAs targeting *TP53* loci, a well‐established tumour suppressor,^17^ were selected and both of the two sgRNAs contain the potential stop codon. Then, sgRNA expression plasmids were transfected into HEK293T cells together with BE3 or Cas9 plasmids. The GFP‐positive cells were harvested 48 hours later using FACS and genomic DNA was extracted to detect the editing efficiency by Sanger sequencing. The results showed both BE3 and CRISPR/Cas9 could edit the target sites. BE3 induced the stop codon while CRISPR/Cas9 produced the indels at the target sites (Figure 1A). To further analyse the knockout effects, we sorted single cells for TP53‐sg1 and got a pure clone (#16) for BE3, and two clones for CRISPR/Cas9 edited cells (#3, #4) (Figure 1B). Then qPCR was performed to determine the mRNA level of *TP53*. Using three pairs of specific primers (termed front, middle and back related to the position of sgRNA), the mRNA level of *TP53,* respectively, showed more than 90 per cent decline (*P* < .001) in BE3‐edited cell lines compared with the wild‐type cells. While in Cas9‐edited cell lines, the gene mRNA level did not show a significant difference compared with the wild‐type cells (Figure 1C). Considering the different mechanism of gene knockout induced by the two systems, we performed Western blot analysis of the three obtained cell lines to demonstrate the knock out effects at protein level, and the results showed no p53 protein was detected, indicating both BE3 and CRISPR/Cas9 could thoroughly knock out the target genes (Figure 1D).

### 3.2 CRISPR/Cas9 results in more genotypes than BE3

**FIGURE 2:**
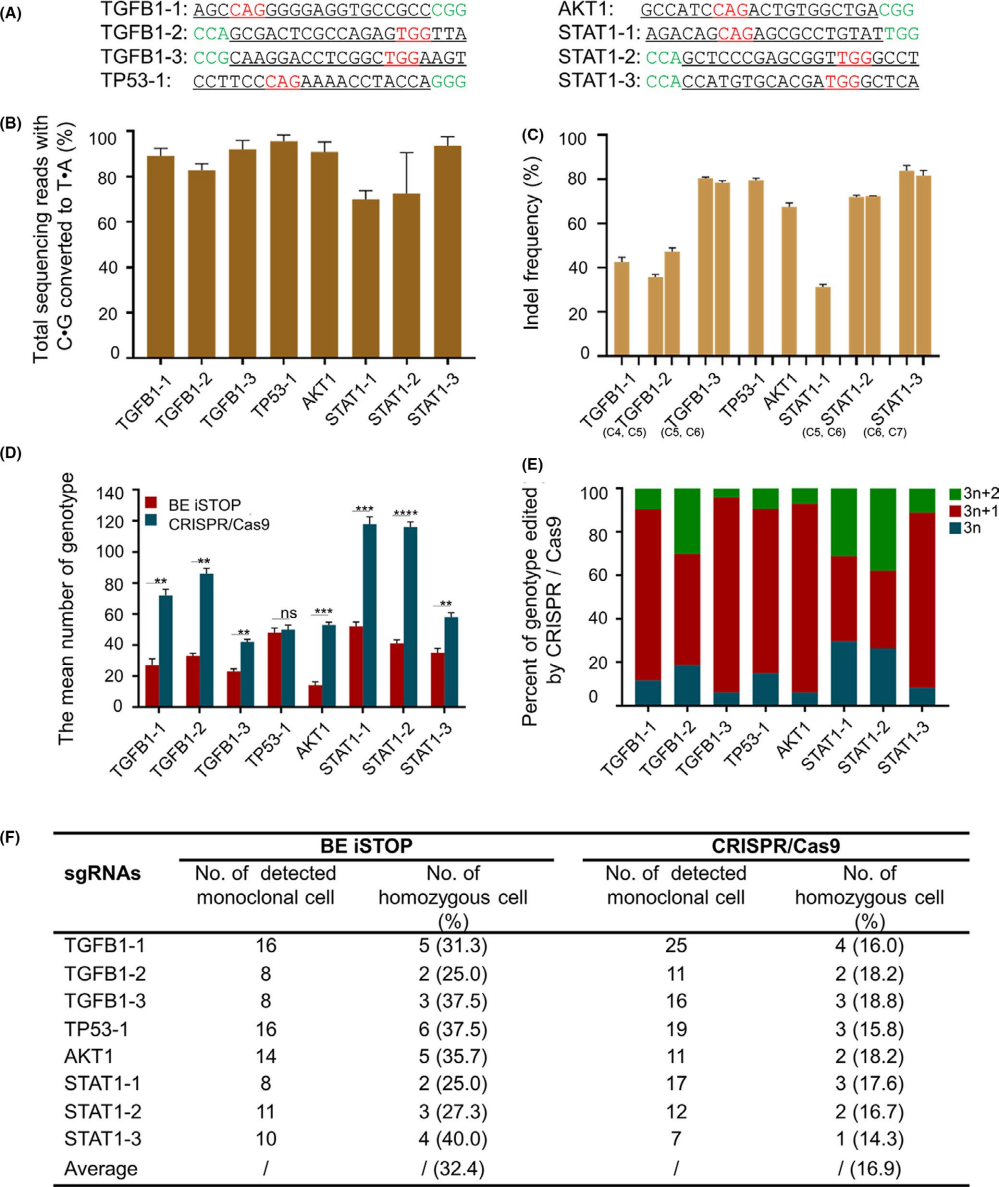
CRISPR/Cas9‐mediated frameshift yields more genotypes than BE‐mediated iSTOP. (A) The used genes and related sgRNA sequences. PAM sequences are highlighted in green. The targeting sequences are underlined. (B) The editing efficiency of BE3‐edited cell populations. Cell populations were the top 25% of GFP‐positive collected by FACS. (C) The indel frequency of Cas9‐edited cell populations. Cell populations were the top 25% of GFP‐positive collected by FACS. (D) The number of genotypes is obtained from BE3‐mediated iSTOP or CRISPR/Cas9‐mediated gene editing. The genotypes were counted after filtering genotypes counted less than 0.1%. Data were analysed by Student's *t* test (**P < .01, ***P < .001, ****P < .0001, ns‐not significant) and shown as mean ± SEM (n = 3 from three independent experiments). (E) Summary of identified monoclonal cells and homozygous cells edited by BE or CRISPR/Cas9. (F) The kinds of genotype for CRISPR/Cas9 edited cells

**FIGURE 3:**
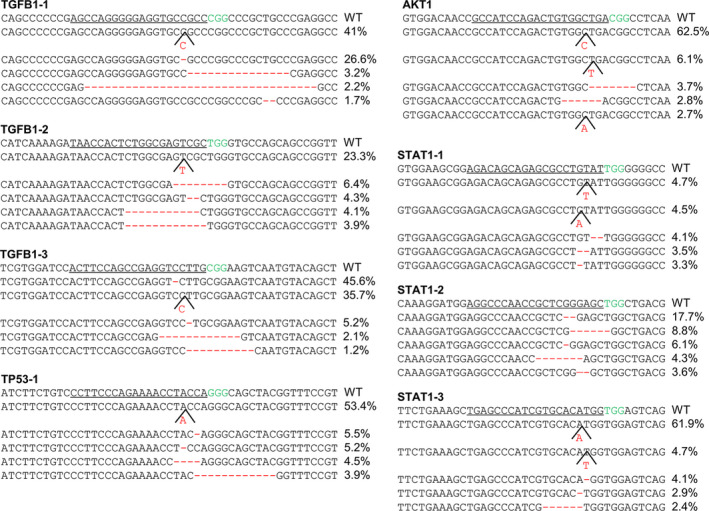
Genotype analysis of target genes edited by CRISPR/Cas9. The top five genotypes were shown. The sgRNA targeting sites are underlined. The PAM sequences are highlighted in green, the insertions and deletions in red

Considering the uncontrollability of Cas9‐mediated genotypes,^6^ we analyse the genotypes obtained from BE3 or Cas9 edited cells. Three sgRNAs for TGFB1, one sgRNA for TP53, one sgRNA for AKT1 and three sgRNAs for STAT1 (Figure 2A) were selected to co‐transfect HEK293T cells with BE3 or Cas9 plasmids. GFP‐positive cells were harvested using FACS, genomic DNA was extracted, and the target sequences were amplified for deep sequencing. Both the BE3‐mediated iSTOP mutation (Figure 2B) and Cas9‐mediated indel (Figure 2C) showed a high efficiency with the specific sgRNAs. The genotypes were counted after filtering genotypes which counted less than 0.1%. The results showed mean number of triplicates in BE3‐edited groups was 27, 33, 23, 48, 14, 52, 41 and 35, respectively. While in Cas9‐mediated editing groups, the paralleled quantities were 72, 86, 42, 50, 53, 118, 116 and 58, respectively (Figure 2D). We summarized the number of detected monoclonal cells, homozygous cells and found the average ratio of homozygote in BE3‐edited groups was 32.4%, while it was about 16.9% in Cas9 groups, suggesting it is easier to get homozygous genotype for BE3 edited cells (Figure 2E). Furthermore, almost all the genotypes induced by BE3 were base substitution (Figure S1). While the insertions or deletions were observed in CRISPR/Cas9 group (Figure 3). Considering the 3n indels may not thoroughly disrupt targeting genes, we divided genotypes into 3n, 3n + 1 and 3n + 2. It is interesting to find the ratio for these three kinds of genotype is distributed randomly. The 3n genotypes counted about 30% in STAT‐1 and STAT‐2 groups (Figure 2F).

### 3.3 Knockout of two neighbouring genes

**FIGURE 4:**
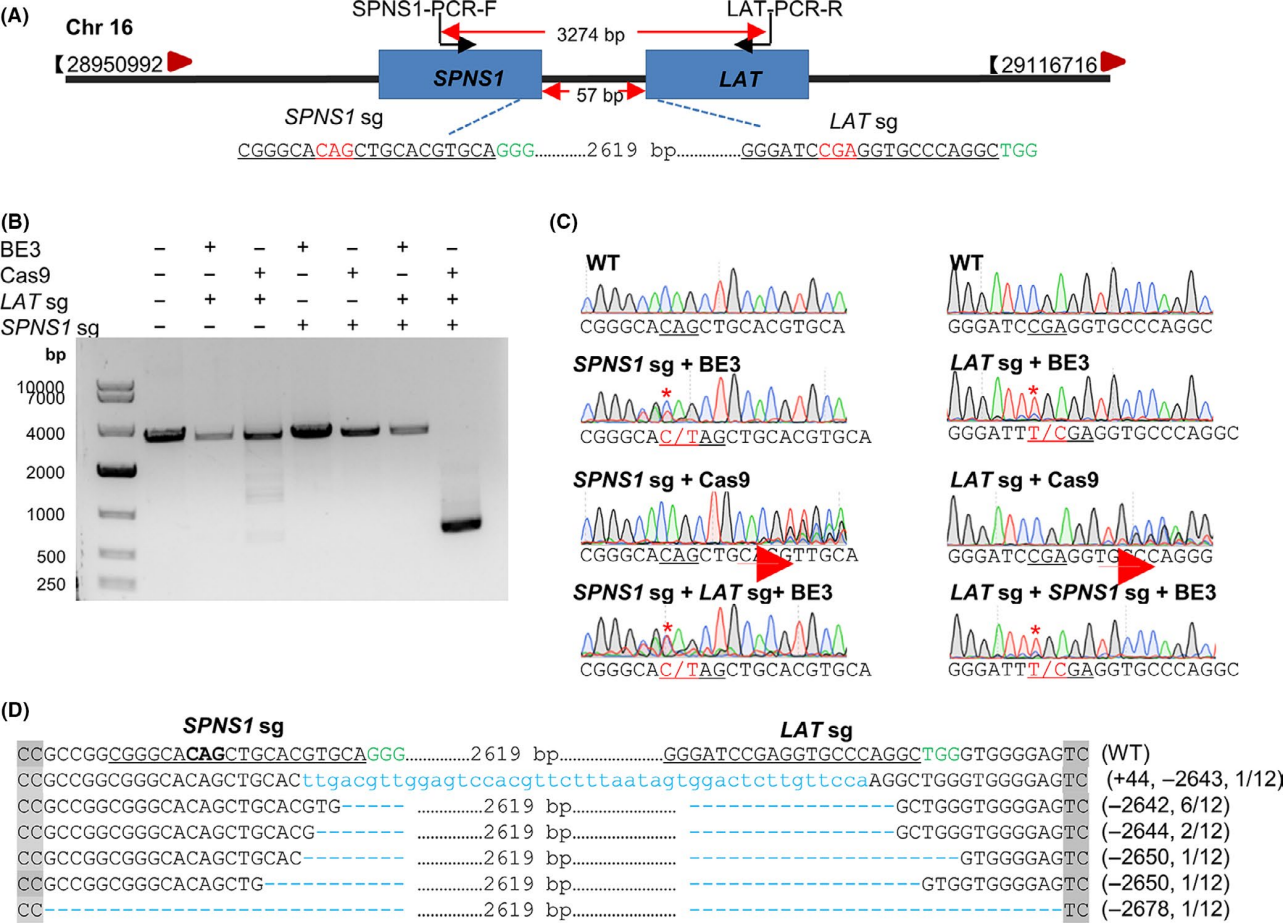
BE‐mediated iSTOP is suitable for multiple genes knockout. (A) Schematic diagram of sgRNAs targeting for *LAT* and *SPNS1* loci. PAM sequences are highlighted in green. sgRNA targeting sites are underlined. (B) PCR products of the targeted region for *SPNS1* and *LAT*. Target regions of *SPNS1* and *LAT* were PCR amplified from sorted GFP^+^ HEK293T cells. (C) Sanger sequencing chromatograms of genome DNA from transfected BE3‐edited HEK293T cells. The red stars indicate the substituted bases. (D) The genotypes of TA clones for CRISPR/Cas9 edited cells. At least 12 TA clones of the PCR products were analysed by DNA sequencing. The sgRNA sequence is underlined; PAM sequences are highlighted in green; the insertions and deletions in blue; deletions (−) and insertions (+)

Considering Cas9/dual sgRNAs may induce fragment deletion,^18^, ^19^ which may affect simultaneous multiple gene disruption, we tried to disrupt two neighbouring genes simultaneously by BE3‐mediated iSTOP. We selected two sgRNAs, respectively, targeting *SPNS1* and *LAT* loci, which are 57 bp away, and the distance of two used sgRNAs is 2619 bp (Figure 4A). When BE3 plasmid was co‐transfected with *SPNS1* sgRNA or *LAT* sgRNA, the amplified products were 3274 bp, as the same size as the control group (Figure 4B). Then, the products were gel extracted for DNA sequencing. The Sanger sequencing results showed that BE3 produced the stop codon for each target site (Figure 4C). The similar size of PCR products was also observed when Cas9 plasmid co‐transfected with *SPNS1* sgRNA or *LAT* sgRNA, respectively (Figure 4B). From the sequencing results, Cas9 produced the indels for each target site (Figure 4C). When *SPNS1* sgRNA and *LAT* sgRNA were co‐transfected with BE3 plasmid, we also obtained a PCR product with 3274 bp. The sequencing results showed both two sites were also efficiently edited (Figure 4C), indicating co‐transfection of BE3 plasmid with two sgRNAs simultaneously produced stop codons for two neighbouring genes. In contrast, for the CRISPR/Cas9 group, a 750 bp fragment was observed instead of the 3274 bp fragment when transfected with *SPNS1* sgRNA and *LAT* sgRNA simultaneously (Figure 4B). It is worth noting that there were also exist other fragments (Figure 4B), implying the knock‐out efficiency did not reach 100%. Then, we recycled the PCR product and analysed the genotype using TA cloning. The results revealed that co‐transfection of Cas9 with *SPNS1* sgRNA and *LAT* sgRNA gave rise to long fragment deletion between the two target sites as expected (Figure 4D). Taken together, BE3‐mediated iSTOP instead CRISPR/Cas9‐mediated frameshift is a good strategy for simultaneously targeting two close loci.

### 3.4 No efficiency difference between stop codons

**FIGURE 5:**
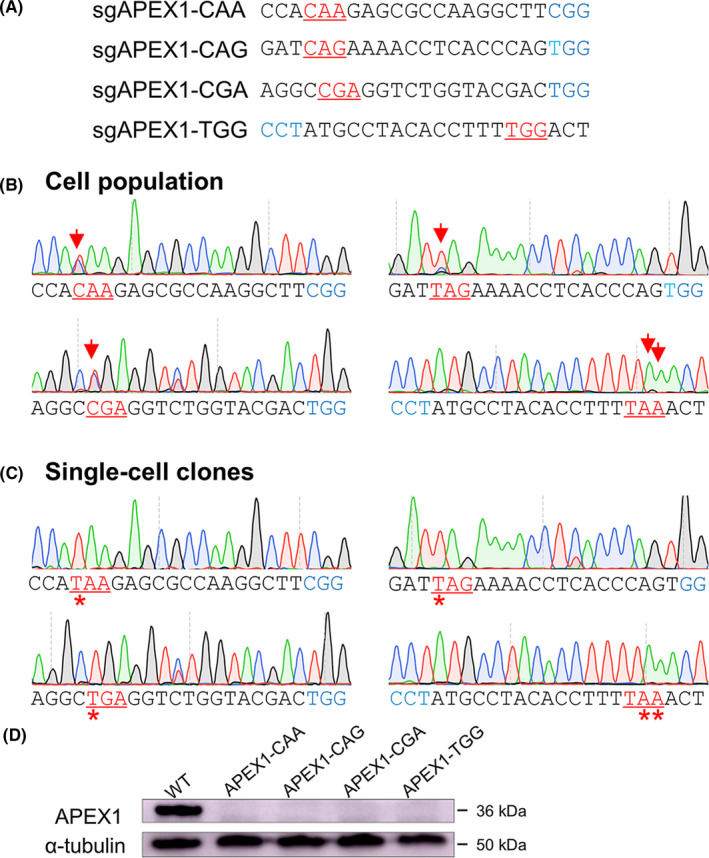
The knockout effects by three potential stop codons. (A) The four sgRNAs for *APEX1* harbouring potential stop codons. (B) The Sanger sequencing showed targeting results of four individual *APEX1* sgRNAs. The cell population indicated the top 20% of GFP‐positive cells sorted from the whole population of cells. The red arrows show the substituted bases. The iSTOP codons are underlined and highlighted in red, and the PAM sequence in blue. (C) The representative Sanger sequences of four *APEX1* knockout single‐cell clones. The red stars show the substituted bases. The iSTOP codons are highlighted in red, and the PAM sequence in blue. (D) The Western blot analysis of APEX1 expression of *APEX1* knockout single‐cell clones

Since there may have different potential stop codons over a gene,^20^ we then asked if there is efficiency difference by different iSTOPs on the same targeted gene. To this end, we designed four sgRNAs to introduce different stop codons for *APEX1* gene (Figure 5A). As expected, the APEX1 protein was successfully knockout in all four kinds of stop codon induced cell lines (Figure 5B‐D), demonstrating three stop codons act identically to disrupt a gene without efficiency difference.

### 3.5 CRISPR/Cas9 has more influence on neighbouring genes than BE3

**FIGURE 6:**
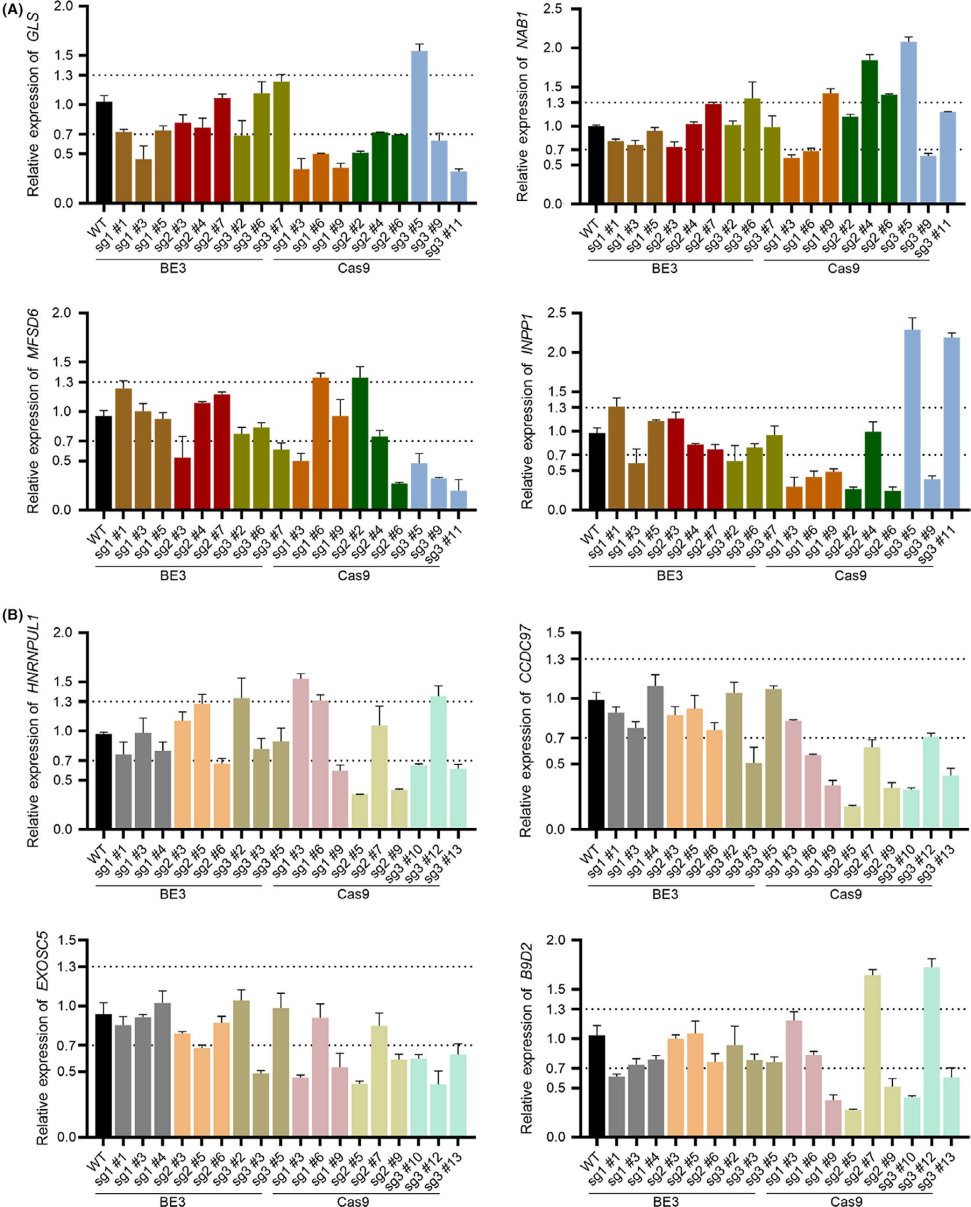
BE‐mediated gene disruption affected neighbouring genes less than CRISPR/Cas9. (A) The relative expression of neighbouring genes around *STAT1* gene. (B) The relative expression of neighbouring genes around *TGFB1* gene. Three sgRNAs for each target gene and three clones for each sgRNA were detected by qPCR. Error bar shown as mean ± SEM (n = 3 from three independent experiments)

To evaluate the influence on neighbouring genes of these two strategies.^8^, ^21^ Three sgRNAs for *STAT1* and *TGFB1*gene and four neighbouring genes for each gene were chosen for the test. The BE3 plasmids or Cas9 plasmids were co‐transfected with corresponding sgRNAs, and three single‐cell clones for each group were detected. Using these modified cell lines, the qPCR analysis was performed to detect the mRNA level of neighbouring and non‐neighbouring genes. Fold change of 1 ± 0.3 was set as threshold. For *STAT1* knockout cell lines, the mRNA level of gene *GLS*, *NAB1*, *MFSD6* and *INPP1* in BE3‐ and Cas9‐edited cells were both affected compared with the WT group, but CRISPR/Cas9 caused much more fluctuations (Figure 6A). For example, in gene of *INPP1* most of the Cas9‐edited clones showed variation out of the scope. Similarly, CRISPR/Cas9 editing had more impact on the mRNA level of gene *HNRNPUL1*, *CCDC97*, *EXOSC5* and *B9D2* (Figure 6B). For non‐neighbouring genes, their mRNA levels had no obvious variation trend, which changed within 1.5‐fold in both *STAT1‐* and *TGFB1*‐knockout cell lines (Figure S2). In summary, BE‐mediated gene knockout showed less side effects on neighbouring genes compared with CRISPR/Cas9 strategy.

## 4 DISCUSSION

Both CBE‐mediated iSTOP and CRISPR/Cas9‐mediated frameshift have been widely used for gene disruption.^22^, ^23^, ^24^, ^25^ Base editing systems provide a precise substitution of the C‐to‐T and A‐to‐G without DSB, thus give less deleterious effects to the genome.^8^, ^15^ Here, we compared the gene disruption mediated by these two strategies carefully. As expected, BE mediated precise base substitution, then yielded fewer genotypes than Cas9‐mediated frameshift, which edits genome uncontrollably, including Cas9‐mediated 3n indels affect the gene disruption. Therefore, significantly fewer genotypes were obtained in BE3 groups compared with CRISPR/Cas9 group. Consequently, it is easy to get isogenic homozygous mutant cell colonies by BE‐mediated iSTOP.

Dual sgRNAs have been used for fragment deletion by Cas9,^18^, ^19^ we tested double knockout of two neighbouring genes and found CRISPR/Cas9 easily delete the large fragment between the two sgRNAs. In contrast BE only induced the stop codon at the target sites, resulting in precise and safe simultaneous disruption of two genes, suggesting BE‐mediated iSTOP is the best choice for multiple genes knockout. Kuscu et al identified that iSTOP strategy had a significant reduction in apoptosis when compared with Cas9 nuclease treatment.^8^ Interestingly, we also found that BE‐mediated knockout had less influence on the relative expression level of neighbouring genes. This may be caused by different level changes by BE‐ or Cas9‐mediated knockout strategies. The BE3 editing cause genotype changing, similarly to single nucleotide polymorphism (SNP), having little effect on the chromosomal structure and neighbouring genes. CRISPR/Cas9 cause significant on‐target mutagenesis, such as large deletions and more complex genomic rearrangements.^6^ Fluctuations in the mRNA level of neighbouring genes may be one of its manifestations.

In summary, we demonstrated the BE‐mediated iSTOP disrupts gene more controllable with fewer side effects.

## CONFLICT OF INTEREST

The authors declare that they have no conflict of interest.

## AUTHOR CONTRIBUTIONS

Huang X and Cui S conceived and designed the project. Dang L, Li G, Wang X, Huang S, Zhang Y, Miao Y and Zeng L performed the experiments. Huang X and Cui S supervised the project. Dang L and Li G analysed the data and wrote the paper.

## Supporting information

Supplementary Material

## Data Availability

All the sequencing data were deposited in the National Omics Data Encyclopedia (NODE) under the project accession OEP000254. The data that support the findings of this study are available on request from the corresponding author.

## References

[1] Doudna JA , Charpentier E . Genome editing. The new frontier of genome engineering with CRISPR‐Cas9. Science. 2014;346(6213):1258096. 25430774 10.1126/science.1258096

[2] Hsu PD , Lander ES , Zhang F . Development and applications of CRISPR‐Cas9 for genome engineering. Cell. 2014;157(6):1262‐1278. 24906146 10.1016/j.cell.2014.05.010 PMC4343198

[3] Sternberg SH , Doudna JA . Expanding the biologist's toolkit with CRISPR‐Cas9. Mol Cell. 2015;58(4):568‐574. 26000842 10.1016/j.molcel.2015.02.032

[4] Komor AC , Badran AH , Liu DR . CRISPR‐based technologies for the manipulation of eukaryotic genomes. Cell. 2017;168(1‐2):20‐36. 27866654 10.1016/j.cell.2016.10.044 PMC5235943

[5] Lukacsovich T , Yang D , Waldman AS . Repair of a specific double‐strand break generated within a mammalian chromosome by yeast endonuclease I‐SceI. Nucleic Acids Res. 1994;22(25):5649‐5657. 7838718 10.1093/nar/22.25.5649 PMC310129

[6] Kosicki M , Tomberg K , Bradley A . Repair of double‐strand breaks induced by CRISPR‐Cas9 leads to large deletions and complex rearrangements. Nat Biotechnol. 2018;36(8):765‐771. 30010673 10.1038/nbt.4192 PMC6390938

[7] Shin HY , Wang C , Lee HK , et al. CRISPR/Cas9 targeting events cause complex deletions and insertions at 17 sites in the mouse genome. Nat Commun. 2017;8:15464. 28561021 10.1038/ncomms15464 PMC5460021

[8] Kuscu C , Parlak M , Tufan T , et al. CRISPR‐STOP: gene silencing through base‐editing‐induced nonsense mutations. Nat Methods. 2017;14(7):710‐712. 28581493 10.1038/nmeth.4327

[9] Aguirre AJ , Meyers RM , Weir BA , et al. Genomic copy number dictates a gene‐independent cell response to CRISPR/Cas9 targeting. Cancer Discov. 2016;6(8):914‐929. 27260156 10.1158/2159-8290.CD-16-0154 PMC4972686

[10] Ihry RJ , Worringer KA , Salick MR , et al. p53 inhibits CRISPR‐Cas9 engineering in human pluripotent stem cells. Nat Med. 2018;24(7):939‐946. 29892062 10.1038/s41591-018-0050-6

[11] Haapaniemi E , Botla S , Persson J , Schmierer B , Taipale J . CRISPR‐Cas9 genome editing induces a p53‐mediated DNA damage response. Nat Med. 2018;24(7):927‐930. 29892067 10.1038/s41591-018-0049-z

[12] Cullot G , Boutin J , Toutain J , et al. CRISPR‐Cas9 genome editing induces megabase‐scale chromosomal truncations. Nat Commun. 2019;10(1):1136. 30850590 10.1038/s41467-019-09006-2 PMC6408493

[13] Tuladhar R , Yeu Y , Tyler Piazza J , et al. CRISPR‐Cas9‐based mutagenesis frequently provokes on‐target mRNA misregulation. Nat Commun. 2019;10(1):4056. 31492834 10.1038/s41467-019-12028-5 PMC6731291

[14] Komor AC , Kim YB , Packer MS , Zuris JA , Liu DR . Programmable editing of a target base in genomic DNA without double‐stranded DNA cleavage. Nature. 2016;533(7603):420‐424. 27096365 10.1038/nature17946 PMC4873371

[15] Billon P , Bryant EE , Joseph SA , et al. CRISPR‐mediated base editing enables efficient disruption of eukaryotic genes through induction of STOP codons. Mol Cell. 2017;67(6):1068‐1079.e4. 28890334 10.1016/j.molcel.2017.08.008 PMC5610906

[16] Korbie DJ , Mattick JS . Touchdown PCR for increased specificity and sensitivity in PCR amplification. Nat Protoc. 2008;3(9):1452‐1456. 18772872 10.1038/nprot.2008.133

[17] Kastenhuber ER , Lowe SW . Putting p53 in context. Cell. 2017;170(6):1062‐1078. 28886379 10.1016/j.cell.2017.08.028 PMC5743327

[18] Cong L , Ran FA , Cox D , et al. Multiplex genome engineering using CRISPR/Cas systems. Science. 2013;339(6121):819‐823. 23287718 10.1126/science.1231143 PMC3795411

[19] Kraft K , Geuer S , Will AJ , et al. Deletions, inversions, duplications: engineering of structural variants using CRISPR/Cas in mice. Cell Rep. 2015;10(5):833‐839. 25660031 10.1016/j.celrep.2015.01.016

[20] Brown A , Shao S , Murray J , Hegde RS , Ramakrishnan V . Structural basis for stop codon recognition in eukaryotes. Nature. 2015;524(7566):493‐496. 26245381 10.1038/nature14896 PMC4591471

[21] Pan Y , Zhang L , Liu Q , et al. Insertion of a knockout‐first cassette in Ampd1 gene leads to neonatal death by disruption of neighboring genes expression. Sci Rep. 2016;6:35970. 27775065 10.1038/srep35970 PMC5075929

[22] Yang G , Zhu TY , Lu ZY , et al. Generation of isogenic single and multiplex gene knockout mice by base editing‐induced STOP. Science Bulletin. 2018;63(17):1101‐1107. 10.1016/j.scib.2018.07.002 36658989

[23] Xu H , Wang B , Ono M , et al. Targeted disruption of HLA genes via CRISPR‐Cas9 generates iPSCs with enhanced immune compatibility. Cell Stem Cell. 2019;24(4):566‐578.e7. 30853558 10.1016/j.stem.2019.02.005

[24] Pankowicz FP , Barzi M , Kim KH , et al. Rapid disruption of genes specifically in livers of mice using multiplex CRISPR/Cas9 editing. Gastroenterology. 2018;155(6):1967‐1970.e6. 30170115 10.1053/j.gastro.2018.08.037 PMC6420307

[25] Kyrou K , Hammond AM , Galizi R , et al. A CRISPR‐Cas9 gene drive targeting doublesex causes complete population suppression in caged *Anopheles gambiae* mosquitoes. Nat Biotechnol. 2018;36(11):1062‐1066. 30247490 10.1038/nbt.4245 PMC6871539

