# Supplementary material for: Comparison of gene disruption induced by cytosine base editing‐mediated iSTOP with CRISPR/Cas9‐mediated frameshift
Source: Cell Prolif. 2020 Apr 29;53(5):e12820. doi: 10.1111/cpr.12820 (PMC7260061; doi:10.1111/cpr.12820)
Supplement: Supplementary file 1 — Supplementary Material [file CPR-53-e12820-s001.docx]

**Supplementary information**

**Comparison of gene disruption induced by cytosine base editing-mediated iSTOP with CRISPR/Cas9-mediated frameshift**

Lu Dang^1^, Guanglei Li^2^, Xinjie Wang^3^, Shisheng Huang^2^, Yu Zhang^2^, Yuanxin Miao^5^, Lisi Zeng^1^, Shuzhong Cui^1, #^, Xingxu Huang^2, 4, #^

*^1^Affiliated Cancer Hospital & Institute of Guangzhou Medical University, 78 Hengzhigang Road, Guangzhou 510095, China*

*^2^School of Life Science and Technology, ShanghaiTech University, Shanghai 201210, China*

*^3^Institute for Brain Research and Rehabilitation, South China Normal University, Guangzhou 510631, China*

*^4^CAS Center for Excellence in Molecular Cell Science, Shanghai Institute of Biochemistry and Cell Biology, Chinese Academy of Sciences; University of Chinese Academy of Sciences, Shanghai 200031, China*

*^5^Jingchu University of Technology, Jingmen 448000, China*

Correspondence: cuishuzhong@gzhmu.edu.cn (S.C.), huangxx@shanghaitech.edu.cn (X.H.)

**
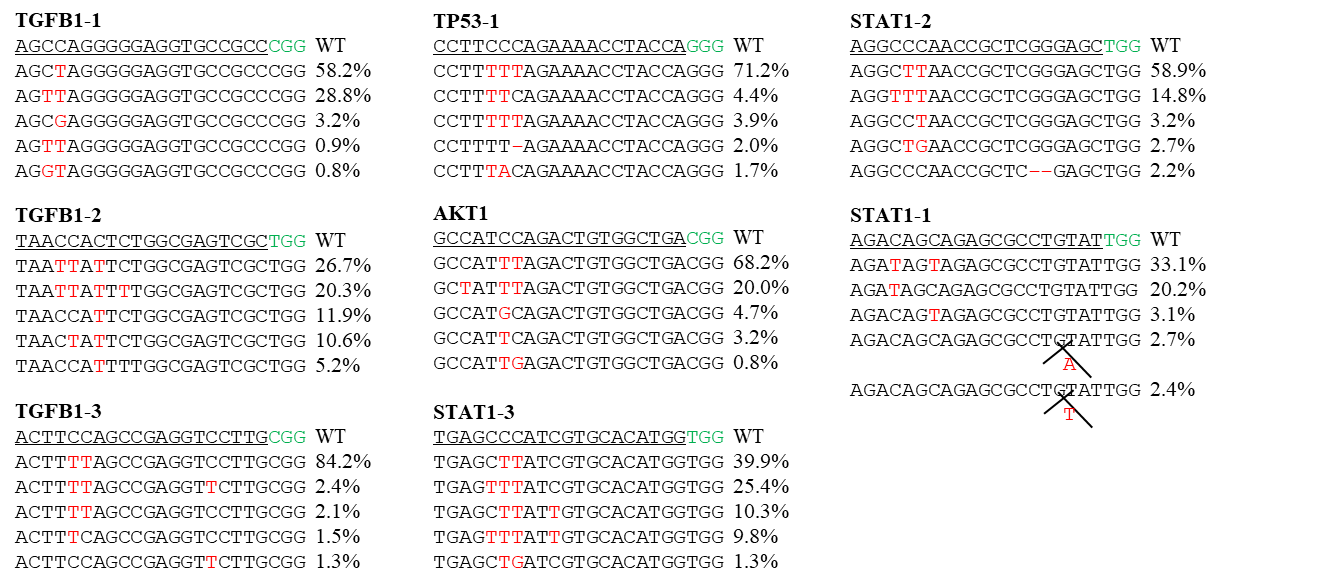
**

**Figure S1. Genotype analysis of the target genes edited by BE3.**

The top five genotypes were shown. PAM sequences were highlighted in green, sgRNA targeting sites are underlined and the substitutions or mutations were highlighted in red.

**
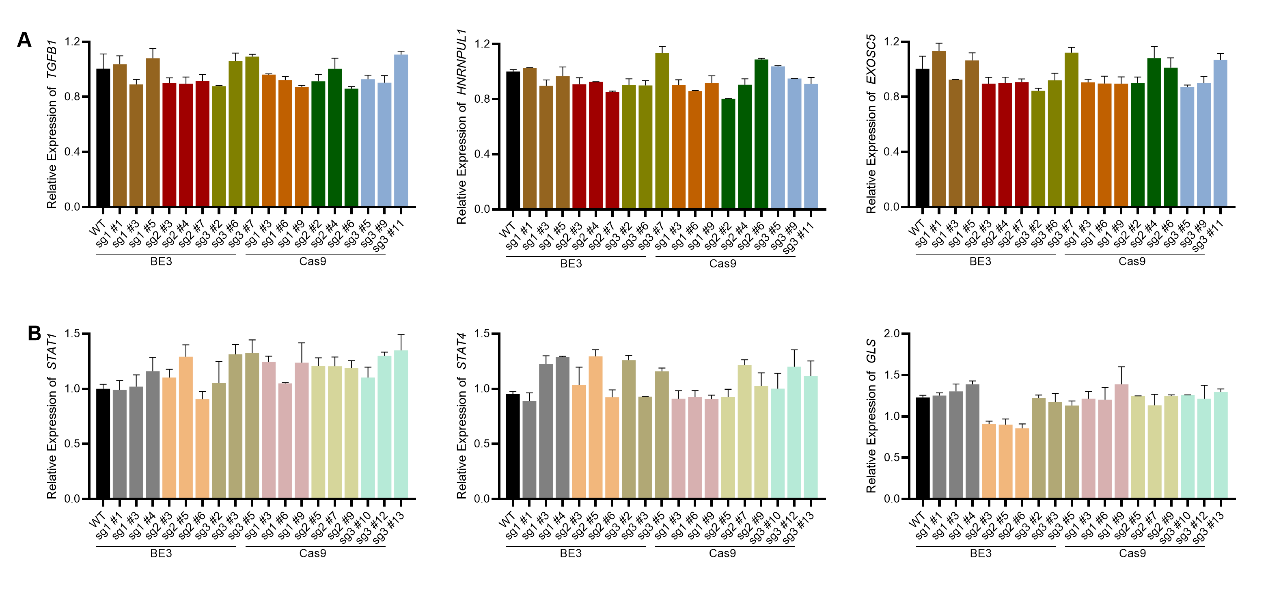
**

**Figure S2. The relative expression level of non-neighboring genes.**

(A) The relative expression level of *STAT1* related non-neighboring genes. (B) The relative expression level of *TGFB1* related non-neighboring genes. Three sgRNAs for each target gene and three clones for each sgRNA were detected by qPCR. These non-neighboring genes also on different chromosome with target genes.

Supplementary Table 1 The sgRNA sequence used in this study.

| **sgRNA name** | **Sequence** |
| --- | --- |
| TP53 sg1 | CCTTCCCAGAAAACCTACCA |
| TP53 sg2 | GCACCAGCCCCCTCCTGGCC |
| STAT1 sg1 | AGACAGCAGAGCGCCTGTAT |
| STAT1 sg2 | GCTCCCGAGCGGTTGGGCCT |
| STAT1 sg3 | CCATGTGCACGATGGGCTCA |
| TGFB1 sg1 | AGCCAGGGGGAGGTGCCGCC |
| TGFB1 sg2 | GCGACTCGCCAGAGTGGTTA |
| TGFB1 sg3 | CAAGGACCTCGGCTGGAAGT |
| AKT1 sg1 | GCCATCCAGACTGTGGCTGA |
| APEX1 sg1 | AGGCCGAGGTCTGGTACGAC |
| APEX1 sg2 | CCACAAGAGCGCCAAGGCTT |
| SPNS1 sg1 | CGGGCACAGCTGCACGTGCA |
| LAT sg1 | GGGATCCGAGGTGCCCAGGC |

Supplementary Table 2 Primer used for PCR amplification.

| **Primer name** | **Primer sequence** |
| --- | --- |
| TP53-PCR-F | CCCATCTACAGTCCCCCTT |
| TP53-PCR-R | AGGGTGTGATGGGATGGAT |
| STAT1-PCR-S1-F | GCTTAGAGCCCCAGTTGAGAAT |
| STAT1-PCR-S1-R | TACAGTCAAACGACACCCCAT |
| STAT1-PCR-S2-F | GGCTAAGCAGTTATCTGAAGGT |
| STAT1-PCR-S2-R | AAATGTTGTGACCAGAGGCTT |
| STAT1-PCR-S3-F | GACCCCAAAGATGCCATGTAT |
| STAT1-PCR-S3-R | AGCAGAGGGGAAAAGAGCAAT |
| TGFB1-PCR-S1-F | TGTGGCTACTGGTGCTGA |
| TGFB1-PCR-S1-R | TCACCGTTGTGGGTTTCC |
| TGFB1-PCR-S2-F | TTCTGGAAGCCTGTTTGG |
| TGFB1-PCR-S2-R | GGTCTCAGCACTTTCACA |
| TGFB1-PCR-S3-F | TGTGGCTTCTATGGTGGTA |
| TGFB1-PCR-S3-R | CCTCTCTGACTTTACTTCTCTT |
| AKT1-PCR-S1-F | CTTCGTGACCCTGAGTGT |
| AKT1-PCR-S1-R | GGACACCTCCATCTCTTCA |
| AKT1-PCR-F | GAGGCTTGGAGAGAGGAA |
| AKT1-PCR-R | GGAGTGAGGATGGCTACA |
| LAT-SPNS1-F | CACTTCTCACCTTCCATTGTC |
| LAT-SPNS1-R | CTGGGTTGTGATAGTCGTCC |

Supplementary Table 3 Primer used for qPCR analysis.

| **Primer name** | **Primer sequence** |
| --- | --- |
| TP53-qPCR-Fr-F | CAGACCTATGGAAACTACTTC |
| TP53-qPCR-Fr-R | GTCTTCAGTGAACCATTGTT |
| TP53-qPCR-Mi-F | GTCATCTTCTGTCCCTTCC |
| TP53-qPCR-Mi-R | GAGTACGTGCAAGTCACA |
| TP53-qPCR-Ba-F | CCTCCTCAGCATCTTATCC |
| TP53-qPCR-Ba-R | TACAGTCAGAGCCAACCT |
| WRAP53-qPCR-F | GACGCATTCACTGGAGAG |
| WRAP53-qPCR-R | GGTTGAAGCCACAGAAGA |
| ATP1B2-qPCR-F | TCTCGGCAACTTCGTCAT |
| ATP1B2-qPCR-R | GGTCACATTCAGGAACTTCA |
| SAT2-qPCR-F | ATATCCTGAGGCTGATTCG |
| SAT2-qPCR-R | TGCTACCAAACAGTGATAGA |
| HNRNPUL1-qPCR-F | TATTACCAGGCAGAACCAAT |
| HNRNPUL1-qPCR-R | GTGCTCCTTGCTTCATCT |
| CCDC97-qPCR-F | CTCTCCAACTTGCTGCTC |
| CCDC97-qPCR-R | TTCCTCGTCACTGTCCTC |
| B9D2-qPCR-F | GCTCCATTTCCAGGTGTG |
| B9D2-qPCR-R | CAACTGTTCTCGCCAACT |
| TMEM91-qPCR-F | GATAGCCTTTGCCGAGTC |
| TMEM91-qPCR-R | GAGTCACTGTCACTGGATG |
| EXOSC5-qPCR-F | GAGGAGGAGACGCATACT |
| EXOSC5-qPCR-R | GAAGGAAGCAGAGCCATC |

Supplementary Table 4 Primer used for deep sequencing.

| **Primer name** | **Primer sequence** |
| --- | --- |
| TP53-dPCR-F | CAGATGAAGCTCCCAGAAT |
| TP53-dPCR-R | GCATTGAAGTCTCATGGAAG |
| STAT1-dPCR-F1 | TGCCTTTAGGAAGTAGTTCA |
| STAT1-dPCR-R1 | TATGTGGTTAGCCAGTCAG |
| STAT1-dPCR-F2 | AGTTGAGGCTAAGCAGTTAT |
| STAT1-dPCR-R2 | GGACCAAAGCAAATGTGTT |
| STAT1-dPCR-F3 | TGAACTTTGGGATGGACATA |
| STAT1-dPCR-R3 | AGCTTCTCTCCCAACATG |
| TGFB1-dPCR-F1 | GACATGGAGCTGGTGAAG |
| TGFB1-dPCR-R1 | ACCTCCTTGGCGTAGTAG |
| TGFB1-dPCR-F2 | AATACAGCAACAATTCCTGG |
| TGFB1-dPCR-R2 | GGGTCCTAGGCAAAGTGA |
| TGFB1-dPCR-F3 | TGTGGCTTCTATGGTGGTA |
| TGFB1-dPCR-R3 | CTTGCTGTACTGCGTGTC |
| AKT1-dPCR-F1 | TGGAACCACGCTTGTGAG |
| AKT1-dPCR-R1 | GGACACCTCCATCTCTTCA |
